# Modular programming for tuberculosis control, the “AuTuMN” platform

**DOI:** 10.1186/s12879-017-2648-6

**Published:** 2017-08-07

**Authors:** James McCracken Trauer, Romain Ragonnet, Tan Nhut Doan, Emma Sue McBryde

**Affiliations:** 1 School of Public Health and Preventive Medicine, Monash University, 99 Commercial Road, Melbourne, 3004 Australia; 2 The Burnet Institute, 85 Commercial Road, Melbourne, 3004 Australia; 3 Department of Medicine, Clinical Sciences Building, the Royal Melbourne Hospital, Parkville, 3050 Australia; 4 Australian Institute of Tropical Health and Medicine, James Cook University, Townsville, 4811 Australia

**Keywords:** Disease transmission, infectious, Tuberculosis, Models, biological, Global health, Software, Tuberculosis, multidrug-resistant

## Abstract

**Background:**

Tuberculosis (TB) is now the world’s leading infectious killer and major programmatic advances will be needed if we are to meet the ambitious new End TB Targets. Although mathematical models are powerful tools for TB control, such models must be flexible enough to capture the complexity and heterogeneity of the global TB epidemic. This includes simulating a disease that affects age groups and other risk groups differently, has varying levels of infectiousness depending upon the organ involved and varying outcomes from treatment depending on the drug resistance pattern of the infecting strain.

**Results:**

We adopted sound basic principles of software engineering to develop a modular software platform for simulation of TB control interventions (“AuTuMN”). These included object-oriented programming, logical linkage between modules and consistency of code syntax and variable naming. The underlying transmission dynamic model incorporates optional stratification by age, risk group, strain and organ involvement, while our approach to simulating time-variant programmatic parameters better captures the historical progression of the epidemic. An economic model is overlaid upon this epidemiological model which facilitates comparison between new and existing technologies. A “Model runner” module allows for predictions of future disease burden trajectories under alternative scenario situations, as well as uncertainty, automatic calibration, cost-effectiveness and optimisation. The model has now been used to guide TB control strategies across a range of settings and countries, with our modular approach enabling repeated application of the tool without the need for extensive modification for each application.

**Conclusions:**

The modular construction of the platform minimises errors, enhances readability and collaboration between multiple programmers and enables rapid adaptation to answer questions in a broad range of contexts without the need for extensive re-programming. Such features are particularly important in simulating an epidemic as complex and diverse as TB.

**Electronic supplementary material:**

The online version of this article (doi:10.1186/s12879-017-2648-6) contains supplementary material, which is available to authorized users.

## Background

The latest estimates for tuberculosis (TB) burden unequivocally identify the pathogen as the world’s leading infectious killer [1]. Despite the consistent claim that rates of disease are declining, the last three Global Tuberculosis Reports from the World Health Organization (WHO) estimate a greater burden of disease than that in the preceding year [1–3]. Although this may be attributable to improvements in case detection and better recognition of the scale of the problem, insufficient funding for both control programs and for research threatens to derail even the modest current progress being made [1, 4]. Despite these obstacles, several technologies are now emerging, including novel diagnostics, medications and treatment regimens [5], while the post-2015 End TB Targets are an ambitious call to action [6]. Therefore, the field of TB control is now in a state of flux, with well-established methods of control competing against new technologies for a share of the limited TB control budgets of highly-endemic countries. Such countries need to understand better the likely epidemiological and economic consequences of programmatic decisions for TB control to guide strategic investment to maximise impact, particularly given that the highest burden countries are almost universally those with the lowest budgets. The recent addition of a financial outcome (catastrophic costs) to the established disease burden targets further emphasises the importance of the economics of such decisions.

Mathematical models are powerful tools to evaluate programs for which evaluation through intervention is impractical, unethical or impossible [7]. Despite significant uncertainties regarding the best approach to structuring and parameterising dynamic models of TB transmission [8], such models are frequently and increasingly used to answer key policy questions in TB control at the national or sub-national level [9, 10]. Moreover, if such models are to be applied repeatedly across multiple regions of the world, they must be able to address the dramatic differences in TB drivers by context – such as HIV in Africa, migration in Western nations and drug resistance in Eastern Europe.

Our recent research has focused on simulating programmatic responses to TB to answer questions of relevance to TB control policy in high-burden countries. Such modelling is important to countries in the cyclical process of evaluating current programs, setting future priorities and planning the TB response. Both countries and funders increasingly view mathematical modelling as a key component of this cyclical process, which is described in detail by a recent publication on the TIME Impact model for TB. The TIME paper describes important principles in using modelling as a programmatic tool, including the need to partner with such countries to support the development of local epidemiological expertise and improve understanding of the epidemic [11]. Here we describe our development of a software platform to achieve these goals and our approach to the particular challenges in simulating the complex and heterogeneous global TB pandemic.

## Implementation

### Past approach

Our earlier TB modelling studies involved the construction of code and compartmental structure appropriate to the question at hand, but with limited capacity for model elaborations to be re-used in subsequent applications – an approach we believe to be common in infectious disease modelling. For example, our work in Western Province, Papua New Guinea incorporated a matrix of population mixing to capture differences between districts of the Province [12], while our modelling in Karakalpakstan, Uzbekistan allowed for mis-assignment of patients according to the extent of drug resistance of the infecting organism [9]. While some elements of our code could be re-used in subsequent applications, the need for extensive re-working of such models for each application was a significant inefficiency.

### Modular development

**Fig. 1:**
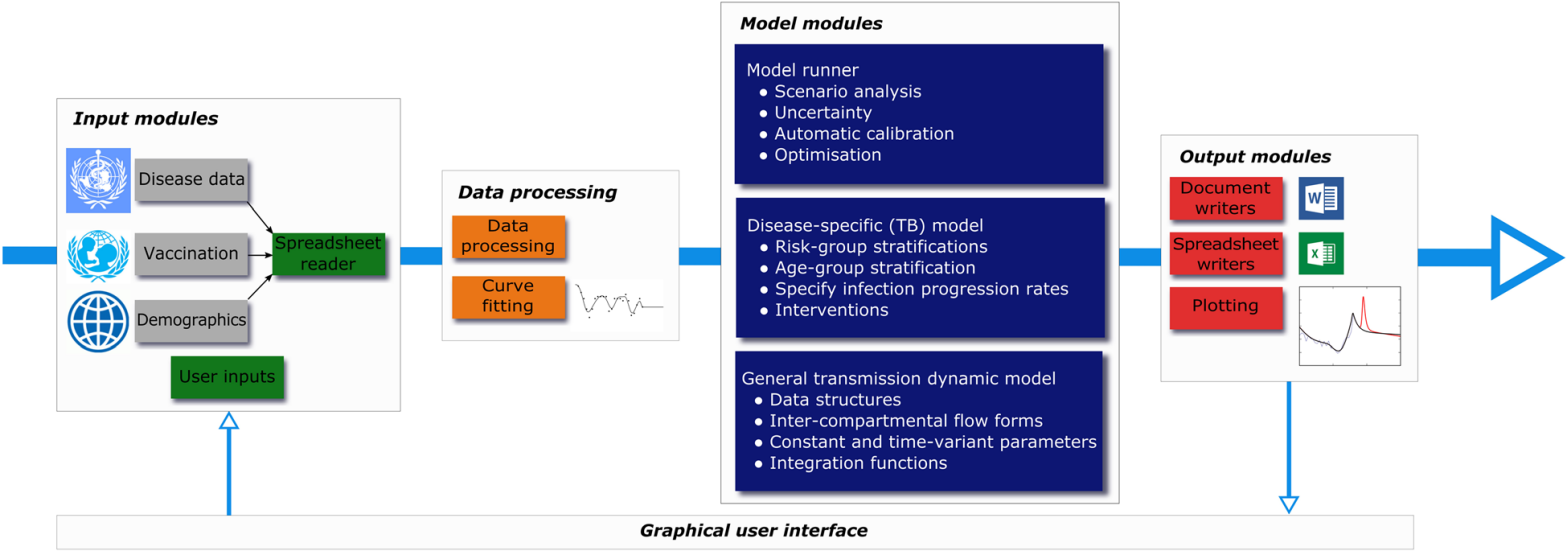
Modular structure of the AuTuMN platform

We fundamentally changed our approach to model development, incorporating principles from computer science and coding in open-source object-oriented Python (version 2.7). This is intended to make our software platform more reliable, testable, reusable and interpretable, as well as to expand the possible functions for which the model can be used and facilitate multi-person programming (via GitHub). The modular structure of our software platform (branded “AuTuMN”) is presented in Fig. 1. After user inputs have been received through the Graphical user interface module, the processes involved in model running proceed in a logical sequence from data processing through model execution to creation and display of model outputs. We used an object-oriented programming approach, with each module (represented by a rectangle in Fig. 1) being a single class. Each such module accepts and produces a consistent set of data structures for the linked modules, to avoid a situation in which adjustments to a single module requires adaptations of other modules. Default settings avoid errors when the user selects a certain option (e.g. age stratification) without specifying necessary associated inputs (e.g. the corresponding age breakpoints).

### Coding syntax

In addition to the modular development, we aimed for our code to be stylistically consistent and adhere to sound basic principles of computer programming. In addition to object-oriented and modular programming, these principles include extensive commenting and explanation of code, using functions wherever possible, using loops to avoid code repetition, employing consistent naming of variables and consistent structuring of data and object attributes. Advantages of employing such principles include ensuring that the code is more readable and interpretable by the multiple contributing programmers, reducing the possibility of errors and allowing functions developed for use in a certain part of the code base to be re-used elsewhere.

### Characteristics of the epidemiological and economic models

**Fig. 2:**
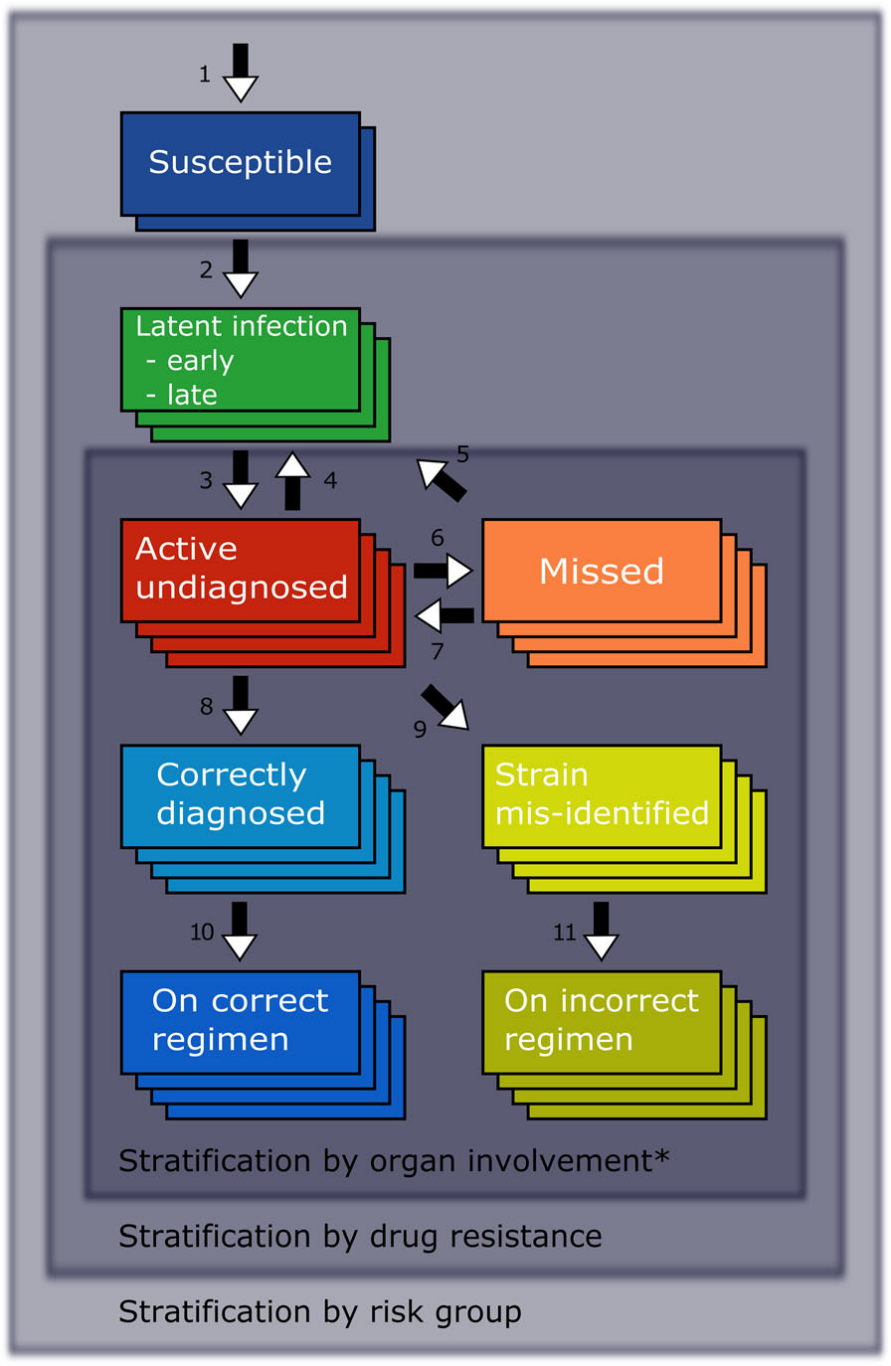
Compartmental structure of transmission dynamic model. Recovery to susceptible compartments after successful completion of treatment, default with return to active disease, death and intervention-related flows are universally implemented but not presented in this Figure. Greater number of overlapping rectangles indicates greater degrees of model stratification, although number of rectangles is arbitrary. Flows presented are: 1, births; 2, infection; 3, progression to active disease; 4 and 5, spontaneous recovery; 6, missed diagnosis due to insensitivity of the diagnostic algorithm; 7, return to care seeking; 8, detection with correct assignment by drug resistance profile; 9, detection with incorrect assignment by drug resistance profile; 10 and 11, commencement on treatment. *“Organ involvement” refers to whether patient has smear-positive, smear-negative or extrapulmonary disease

The general compartmental structure of the TB transmission dynamic model is presented in Fig. 2 and described in Section 1 of Additional file 1. As model stratification is intended to be variable by context and is determined by user inputs, it is not possible to present a universal flow diagram or list of differential equations. However, the structure presented depicts most of the main transition flows between compartments that are universal to all applications of the model. The epidemiological principles of model construction have been described in several of our previous publications and are retained through all AuTuMN country applications. These include: partial vaccine efficacy [13], waning risk of progression to active disease with time from infection [14, 15], a three year average duration of active disease [16], distinguishing the process of detection from that of determining the drug resistance profile [9], amplification of drug resistance with default from treatment [14, 17] and a partial reduction in transmissibility for organisms with greater levels of drug resistance [9, 12].

A logistic cost-coverage function links spending on programmatic interventions to coverage and subsequent epidemiological impact. We consider start-up costs as additional fixed costs for programs absent from the baseline scenario, because we are frequently comparing well-established interventions (e.g. BCG vaccination) against the implementation of newly proposed technologies (e.g. molecular diagnostics). Therefore, we assume a maximal cost-coverage gradient at zero spending, after start-up costs have been applied.

## Results

Earlier iterations of our model (coded in Matlab™ without adherence to all the programming principles described above) were applied to inform TB control programs in five contexts: Western Province of Papua New Guinea [12], Karakalpakstan in Uzbekistan [9], and India, China and South Africa through the TB-MAC-coordinated “Targets” exercise [10, 18]. With support from the Global Fund to Fight AIDS, TB and Malaria, we were then commissioned to undertake policy-relevant modelling for the National Tuberculosis Programs (NTPs) of three further countries: Fiji, the Philippines and Bulgaria. Reports on the epidemiological and economic results for each of these three applications have been submitted to the NTPs of each country and we also aim to publish these in peer-reviewed journals in the coming months in collaboration with our country partners, including detailed exposition of the epidemiological assumptions and parameter values made in each. The software allows for immediate adaptation of many features of the transmission model to each context through the Graphical user interface, such as addition of risk groups or strains and changes to the purpose of the simulation. The “General transmission dynamic model” module has been released as open-source software (see https://github.com/popdynamics/popdynamics), while a repository that presents the framework of each platform module is also available (see https://github.com/jtrauer/AuTuMN_framework). These repositories include code, dependencies and detailed user documentation. The characteristics of each module are presented in Table 1, with examples provided in the AuTuMN_framework repository.

**Table 1:** Characteristics of modules

*Module*	*Characteristics*
Graphical user interface	Accepts user inputs to determine: • Country to be simulated • Purpose of simulation ○ Uncertainty, optimisation, automatic calibration, scenario analysis ▪ Scenarios required (uncertainty or scenario analysis only) ○ Saving and loading of previous simulations • Compartmental model structure, including optional elaborations ○ Multiple health systems differing by quality of care ○ Amplification of drug resistance with treatment default ○ Mis-assignment of drug resistance status of infecting organism ○ Sub-populations and risk groups ○ Heterogeneous mixing between population groups • Other methodological aspects of run ○ Integration method ○ Integration time step ○ Fitting method of parameters to loaded data ○ Epidemiological and economic parameters • Outputs required
Spreadsheet reader	Reads data according to country selected in Graphical user interface Accepts original format of spreadsheets as input (currently all Microsoft Excel™) Converts data to consistent format (Python dictionary with years as keys and data entries as values) Spreadsheets read: • Data on aggregate TB burden and programmatic response, WHO [45] • BCG vaccination coverage, UNICEF/WHO [46] • Demographic variables, the World Bank [47]
Data processing	Creates data structures with a format interpretable by the Model modules • Combines and reconciles external inputs from Spreadsheet reader with user inputs from GUI • Calls Curve fitting module to fit functions to reconciled data structures • Derives parameters determined by multiple input parameters ○ e.g. for comorbidities leading to an increased progression rate (such as diabetes) the risk group-specific progression rate is calculated by multiplying the age group-specific progression rate by the relative progression rate attributable to the risk factor
Curve fitting	Derives polynomial spline functions to represent parameters (often interventions) scaling over time (See Fig. 3) • Fits to input data of dictionaries with keys time (in years) and values parameter values (often intervention coverage as a proportion) • Intervention values remain constant into the future from most recent parameter value ○ That is, the default behaviour (baseline scenario) is all interventions frozen at this value
Model runner	Creates and runs model objects according to the purpose selected in the Graphical user interface • For scenarios, runs baseline model followed by each requested scenario with interventions as requested • For uncertainty, iteratively runs model, updating uncertainty parameters between each run ○ Currently uses a Metropolis-Hastings algorithm ○ Priors are estimated from the distributions of included epidemiological parameters ○ Posteriors are estimated from a comparison of outputs to WHO data • For automatic calibration (an extension of uncertainty), iteratively runs model, updates model parameters, starting populations and other epidemiological parameters • For optimisation, estimates epidemiological outputs from proportionate allocation of funding across programs given a certain funding envelope ○ Currently uses SLSQP from the “minimize” function of the scipy.optimize package ○ Considers proportional funding to interventions to be the bounds ○ Considers the function to be minimised to be the epidemiological output of interest (usually incidence or mortality) when scenarios are run with varying funding allocation from a fixed/calibrated baseline
Disease-specific (TB) model^b^	Defines stratifications and their interaction by: • Age • Comorbidity and/or population risk group • Organ involvement (smear-positive pulmonary, smear-negative pulmonary and extrapulmonary)^a^ • Drug resistance of infecting strain^a^ • Health system quality^a^ Sets inter-compartmental flows, for: • Ageing • Natural progression through stages of infection and disease • Detection (by each stratum of health system quality, if applicable) • Drug resistance status assignment by health system Implements interventions selected from scenarios requested in Graphical user interface • Estimates economics of interventions using logistic cost-coverage curves and population sizes
General transmission dynamic model	Defines fundamental structures of transmission dynamic model which are not pathogen-specific (i.e. components common to any deterministic, compartmental, ordinary differential equation-based model of population-level infectious disease transmission), including: • Compartments • Inter-compartmental flows • Fixed parameters • Variables to be updated at each integration time step
Output modules	Creates Word™ and Excel™ tables of epidemiological and economic outputs and graphical (PNG) figures to illustrate: • Compartmental model structure (using “graphviz” repository) • Time-variant parameters, including fit to input data (see Fig. 3) • Other illustrations of epidemiological implementation of interventions ○ e.g. visualisation of matrix of population mixing • Aggregate model outputs compared to Global TB Report estimates, for: ○ Scenarios ○ Uncertainty (see Fig. 4) • Model outputs by risk groups • Optimised funding distribution across interventions

^a^Note that not all types of stratification apply to all compartments (e.g. susceptible population not stratified by drug resistance of infecting organism), see Fig. 2. ^b^This module is not a stand-alone class, but instead inherits general methods from the General transmission dynamic model module, adding TB-specific methods to the class. Abbreviations: TB, tuberculosis; PNG, portable network graphics; UNICEF, The United Nations International Children’s Emergency Fund; WHO, World Health Organization.

Examples of our coding approach are presented in Table 2 and Section 2 of Additional file 1: (Table S1). Note the consistent naming conventions. For example, parameter name strings are separated by underscores that describe first the source of the parameter (e.g. disease specific = ‘*tb*’, programmatic = ‘*program*’), then the nature of the parameter (e.g. rate = ‘*rate*’, proportion = ‘*prop*’), then the meaning of the parameter (e.g. spontaneous recovery = ‘*recover*’, untreated death = ‘*death*’) and lastly the stratum to which the parameter applies if applicable (e.g. smear-positive/negative/extrapulmonary status = ‘*organ*’). As the source, meaning, units and applicability of each parameter are implied by its name string, there is less need for detailed comments to explain the meaning of each parameter at each code line. Also, the division of the code into functions responsible for a very small component of the entire platform’s function limits the potential for errors and enables changes to be made that only affect one component of the platform. For example, changes to progression through stages of active disease could be adjusted without the need to check that all inter-compartmental flows have been appropriately coded and that flows are conserved.

**Table 2:**
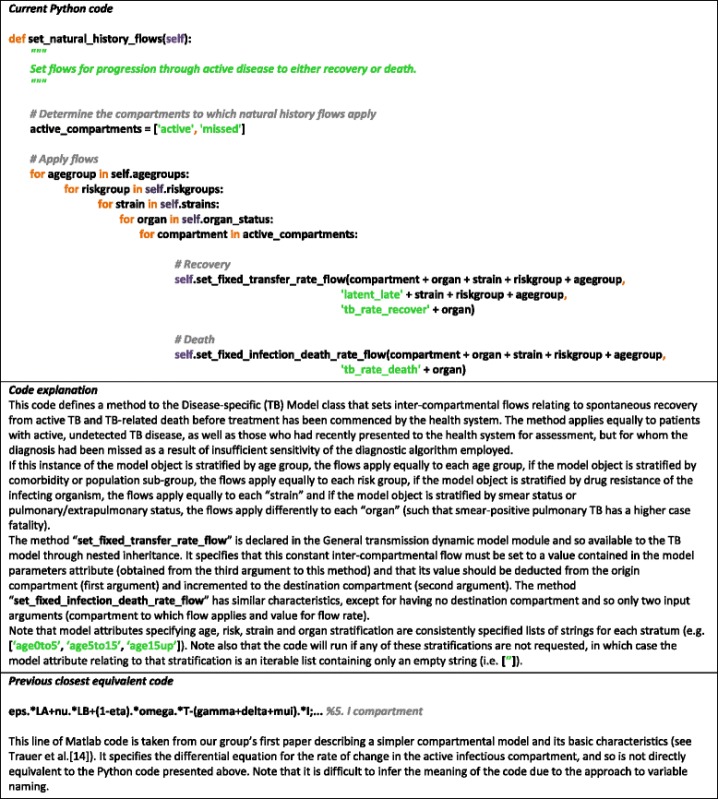
Illustration of approach coding

**Fig. 3:**
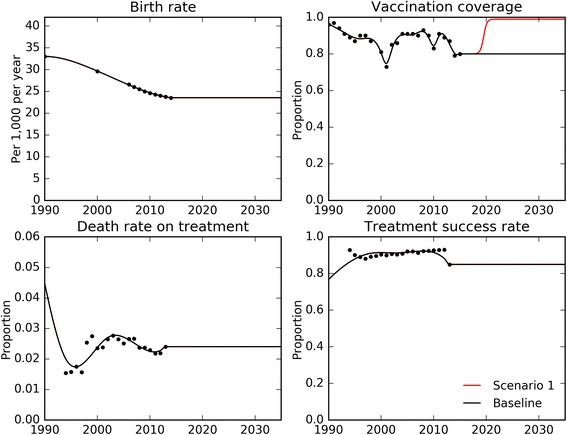
Fitting of time-variant parameters to data. Black dots, loaded data for the Philippines from – World Bank (birth rate), UNICEF (vaccination coverage) and Global TB Report (death rate on treatment and treatment success rate). Solid lines, time-variant parameter functions – black, baseline scenario; red, example scenario of scale-up of vaccination coverage

**Fig. 4:**
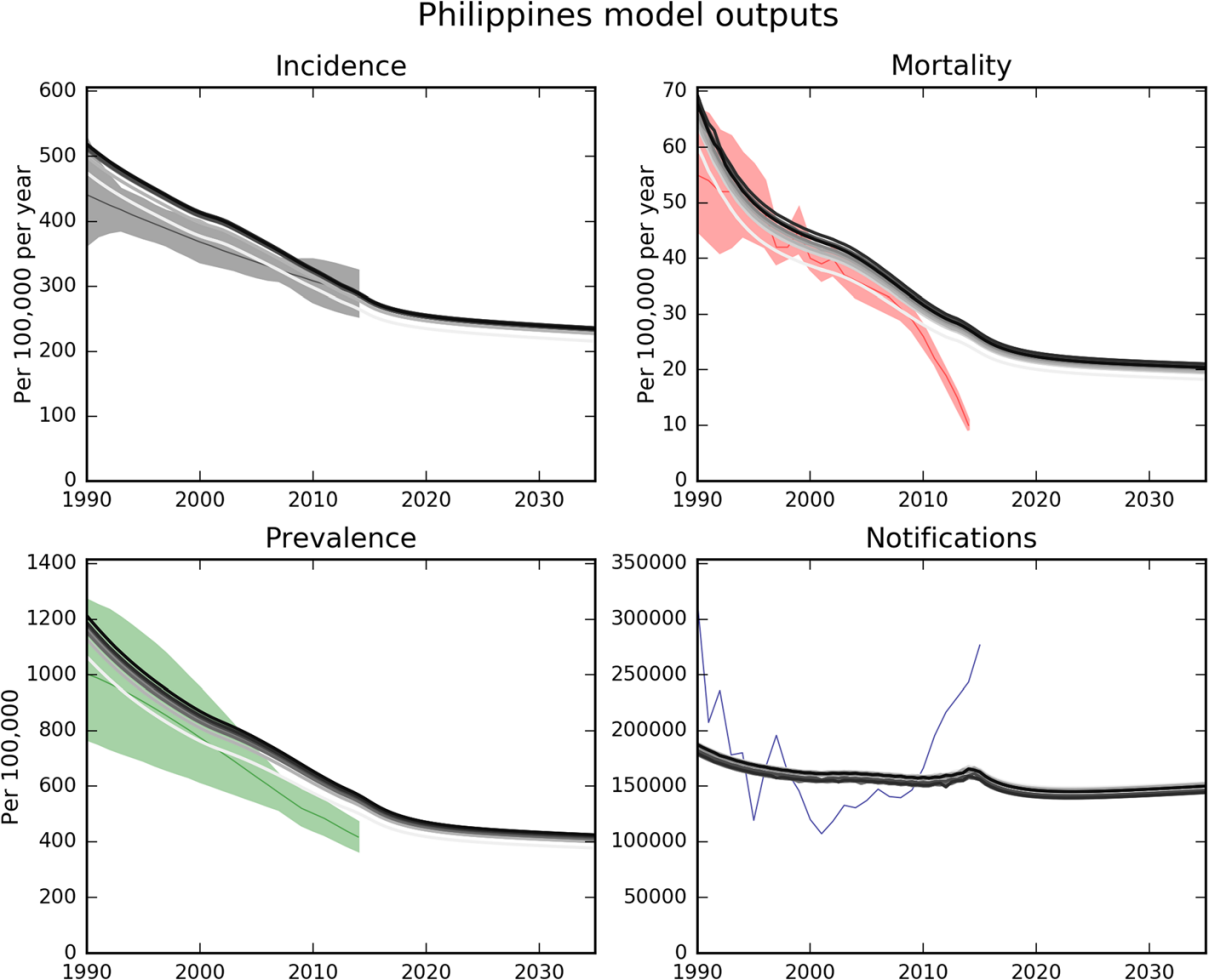
Visual outputs from model calibration to data for epidemiological indicators. Data are from Global TB Report 2016 for the Philippines. Progressively darker parallel grey lines, successive model runs accepted by the Metropolis-Hastings algorithm; coloured shaded areas (where presented), calibration data uncertainty ranges; thin central coloured lines in shaded areas, calibration data point-estimates. This example calibration is to reported incidence data from 1990 to 2016 with weighting to emphasise calibration to more recent years for which data are available, with three uncertainty parameters (effective contact rate, duration of untreated TB, case-fatality of untreated TB), with values starting from values found during a manual calibration

Figure 3 illustrates our approach of automatically fitting time-variant parameter values to data loaded through the Spreadsheet reader module and an example of variation in a programmatic parameter (vaccination coverage) for scenario simulation. After setting time-variant parameters, a Metropolis-Hastings algorithm is used to vary uncertain (time-constant) parameters in order to calibrate the model to epidemiological data on disease burden, with the progression of parameter values displayed through the Graphical user interface in real time. Progression of model outputs over consecutive runs can also be displayed overlaid on calibration data from the Global TB Report displayed as shaded areas (Fig. 4). A similar approach to parameter variation is also used to quantify uncertainty in future predictions.

**Fig. 5:**
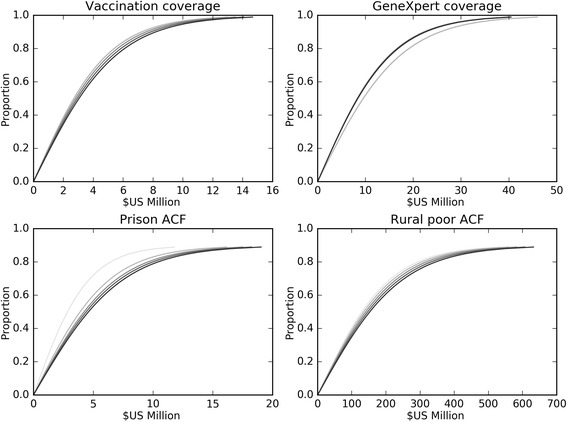
Cost-coverage curves for example scenarios. Cost-coverage curves for four example scenarios in the Philippines. ACF, active case finding. Progressively darker shading indicates progression in cost-coverage relationship over time, in five year increments from 2015 to 2035 (as relationships are dependent on the size of the population targeted by each intervention)

Figure 5 presents examples of our approach to implementing logistic cost-coverage curves for interventions and hence estimating costs over time for each program. The logistic function links an intervention’s coverage to its associated cost and is presented in detail in Section 3 of Additional file 1. This function incorporates variables such as population sizes that are updated at each iteration of the model integration so that the economic and epidemiological modules interact continuously. The cost-coverage curves can be used in two directions: costing of programs when coverage values are specified, and adjustment of coverage based on input costs.

Outputs from our model include assessment of the baseline epidemiology, such as reconciling data for incidence, mortality, prevalence and notifications or explaining why such indicators appear mutually inconsistent. We also present estimates of the likely impact of planned or considered changes to the programmatic response based on our scenario simulations. Next, the epidemiological and economic differences between the baseline scenario and these counter-factual scenarios are then used for cost-effectiveness analysis. Last, an optimisation algorithm allows for minimisation of disease burden indicators over a specified time horizon, with such indicators including incidence, prevalence, mortality and proportion of TB multidrug-resistant in the overall population or sub-groups, as well as any weighted combination of these indicators. The current approaches to calibration and optimisation are described briefly in Table 1. However, given that each procedure is called from only one function of the Model runner module, alternative techniques could easily be substituted.

## Discussion

We present the development of our novel software platform and mathematical model of TB transmission. The significant change in computer programming philosophy that we adopted to tailor it to TB control simulations helps improve the flexibility, reliability and reusability of our software. Our modular approach allows for compartmentalisation of the software’s functions, in order to improve these characteristics of the tool and allow for a broader range of functionality, including stratification by age, risk group, strain and organ involvement, historical understanding of the epidemic, uncertainty, automatic calibration, cost-effectiveness and optimisation. Dynamic models of infectious disease transmission have the major advantage that they are able to capture the non-linear positive feedback loop of greater disease burden leading to more transmission and, in turn, to greater disease burden. However, such models must be fit for purpose and the challenges in developing a realistic tool for simulating TB transmission are considerable. Although mathematical modellers have long used computer programming to produce numerical simulations of infectious disease transmission, this is most often undertaken by small groups of researchers or individual academics and so is often limited to analyses whose end result is expected to be a single journal article.

Model flexibility is critically important in simulating the TB epidemic across multiple settings, because of the heterogeneity in drivers of the TB epidemic – arguably more than for any other disease. For example, HIV coinfection is a critical driver of the high rates of disease in sub-Saharan Africa [19, 20], drug resistance reaches alarming levels in many countries of Eastern Europe [21, 22] and the Asia-Pacific Region is characterised by a huge absolute caseload and a significant proportion of care delivered outside of the public sector [1, 23]. Therefore, the characteristics of the epidemic are dramatically different in these settings, as are the programmatic interventions that policy-makers from these regions wish to understand better. Moreover, the epidemiology of TB immunity, transmission and progression differs dramatically according to several population strata, adding further to the degree of model complexity necessary to capture the epidemic realistically. Such strata include age groups, which affect immunity following vaccination [24], infectiousness [25] and rates of progression from infection to active disease [15]. Similarly, comorbid conditions that affect rates of progression from infection to active disease can have major epidemiological effects, such as HIV [26] and diabetes [27], while the impacts of factors such as poverty and overcrowding are more difficult to quantify but likely to be equally important [28]. Further, patients with smear-negative pulmonary or extrapulmonary disease are known to have significantly lower infectiousness than smear-positive pulmonary disease [29] and treatment outcomes differ markedly according to the drug resistance of the infecting organism [30].

These differences are important to TB models; both to accurately capture the underlying epidemiology and because interventions often act differentially across such disease categories. However, simultaneous model implementation of several such factors can be challenging and lead to high levels of complexity, as the number of compartments to track may scale multiplicatively with the number of stratifications included. Therefore, function-based code that consistently and automatically implements such strata into models regardless of their compartmental structure and other stratifications, add much to the flexibility, reliability and reusability of the software platform. For example, ageing rates between sequential age groups are set automatically once the user has requested a set of breakpoints between age groups.

Tuberculosis is an ancient disease, as well as being a slow-moving epidemic due to the propensity of the infecting organism to re-activate after many years to decades of dormancy [31]. Therefore, accurately capturing the historical dynamics of the disease is of greater importance than for many acute infections, as interventions and dynamics are dependent on disease burden many decades earlier and can be markedly affected by demographic trends. In our analyses, we have consistently found the size of the latent pool to be a key driver of the future disease burden, and that this pool of infection limits the effectiveness seen with interventions directed at active cases alone. To address this issue, all our analyses commence from many decades to centuries into the past and aim to capture the dynamics of the TB response from the point at which they commenced. Time-variant parameters representing this response are fit to freely available officially reported data, and a zero value is added automatically at the time the technology first became available. Similarly, loading of disease burden outcomes from the WHO is important to ensure that model calibrations capture the progression of the epidemic over the course of the time period for which data are available. The General transmission dynamic model module is intended to apply equally to any infectious disease and examples are provided for measles, influenza and TB. The issues specific to ancient, slow-moving endemic infections and TB in particular have partially informed the broader modular development of our tool, although most of the modules could be adapted to any infection. We aim to release further modules as possible, such as the Curve fitting and Spreadsheet reader modules, and to extend the capability of the General transmission dynamic model. However, given the major differences between interventions requested, population-level risk factors and epidemiological context in each country, we make significant adaptations to the Disease-specific model module at each application, and so have no short-term plan to release this module.

A number of modular software platforms exist to estimate future burden of disease. Perhaps best known is the Spectrum suite of models, which simulate population demographics and disease progression, although these are not necessarily underpinned by transmission dynamic models [32, 33]. For influenza, complex microsimulation-based models underpinned by realistic synthetic population structures and explicit transmission dynamics have been made available to policy-makers [34, 35]. Models designed to simulate strategies to combat the huge global burden of HIV have also proliferated, and include the Asian Epidemic Model [36, 37], Optima [38] and the Estimation Projection Package [39–41]. For TB, transmission dynamic model-based tools have been tailored to consider specific interventions for control, such as active case finding [42] and diagnostic strategies [43, 44]. However, such tools are not readily adaptable to consider a broader range of interventions or to compare interventions that act at very different points in an individual’s journey through infection, disease, detection and treatment. A more general TB modelling tool that has been used across several countries worldwide is TIME Impact, which incorporates a transmission dynamic model and is integrated into the Spectrum suite of modelling tools [11]. The most important epidemiological advances of AuTuMN by comparison to TIME relate to flexibility. TIME describes a fixed approach to latency (single latent compartment with bypass), drug-resistant strains (MDR-TB and DS-TB), age groups (5-year age bins), organ status (smear-positive and smear-negative) and comorbidities (HIV). By contrast, AuTuMN permits greater versatility in relation to each of these epidemiological stratifications, while additionally incorporating uncertainty, calibration and optimisation capacity through the Model runner module.

Complicated modelling tools have the disadvantages of increased computational expense (i.e. processing time), potential for errors and increased difficulty for the user to understand and interpret all the dynamics operating within a particular simulation. We minimise the potential for coding errors through consistent coding, minimisation of code repetition and built-in checks throughout our modules. Although we frequently use a highly stratified version of AuTuMN, involving hundreds to thousands of compartments, some or all stratifications can immediately be removed to facilitate the user’s understanding of the underlying epidemiological processes at play. Also, we build in intermediate outputs to model running, in order to facilitate understanding of such processes, such as mixing matrix diagrams and population sizes by sub-group. Despite the rapidly increasing speed of modern computers and methods for decreasing computation time, computation time remains an issue in our model, particularly for uncertainty calculations. Whereas optimisation and scenario analyses typically require the full model to be run forward over the simulation window from a single baseline run (typically twenty to thirty years), uncertainty requires repeated runs from the start of the simulation (typically several decades to centuries). For example, explicit Euler model integration from the start of simulation time with three organ status strata, two strains (DS-TB and MDR-TB), two health care strata differing by quality and five population risk groups takes around 40 s for a full run and ten seconds for each additional scenario run on a standard personal computer (Intel i7 2.6GHz processor, 8 GB RAM). Run-time scales approximately multiplicatively with the number of stratifications for each type of stratification, while the time taken for initialisation remains approximately unchanged at around 7 s.

## Conclusions

We employed several principles of software engineering to develop a robust and flexible tool to support TB control decisions. The tool is underpinned by a transmission dynamic model and is rapidly adaptable to capture many of the complexities of the TB epidemic across a range of settings and the interventions available in the fight against this critically important infectious disease. Such tools are of increasing importance as TB emerges as the world’s leading infectious threat, ambitious new targets for its control have been set and old technologies vie with new tools for limited budgets.

## Availability of data and materials

The code for the “General transmission dynamic model” module has been released as open-source software, including the full code, dependencies and detailed user documentation.

**Project name** popdynamics.

**Project home pages** https://github.com/popdynamics/popdynamics, https://github.com/jtrauer/AuTuMN_framework

**Operating system** Platform independent.

**Programming language** Python version 2.7.

**Other requirements** numpy, scipy, graphviz.py, graphviz binary on path: http://www.graphviz.org/, matplotlib.

**License** Open source.

## Additional file

Additional file 1: Notes on structure of epidemiological model, further code examples and notes on economic model. (DOCX 1113 kb)

## Abbreviations

ACF: Active case finding
AuTuMN: The Australian Tuberculosis Modelling Network (see www.tb-modelling.com)
BCG: Bacille Calmette Guérin
HIV: Human immunodeficiency virus
PNG: Portable network graphics
TB: Tuberculosis
TIME Impact: A new user-friendly TB model to inform TB policy decisions, trade mark (see https://bmcmedicine.biomedcentral.com/articles/10.1186/s12916-016-0608-4)
TM: Trade mark
UNICEF: United Nations International Children’s Emergency Fund
WHO: World Heath Organization
