# Supplementary material for: Modular programming for tuberculosis control, the “AuTuMN” platform
Source: BMC Infect Dis. 2017 Aug 7;17:546. doi: 10.1186/s12879-017-2648-6 (PMC5547473; doi:10.1186/s12879-017-2648-6)
Supplement: Additional file 1: — Notes on structure of epidemiological model, further code examples and notes on economic model. (DOCX 1113 kb) [file 12879_2017_2648_MOESM1_ESM.docx]

## Supplementary Material

### Section 1 Detail of TB model structure

The compartmental structure of our TB model is variable and adapted to the country at hand. However, this section describes the principles by which it is structured. Full explanations of the rationale and evidence for these structures, along with examples of these compartmental approaches are provided in our previous publications (summarised briefly above in “Characteristics of the epidemiological model” in the Methods section of the main text).

As we have stressed, AuTuMN is highly flexible, such that many of the features described below can be included or excluded as per the user’s preference. Therefore, we present the most commonly used configurations, but will describe our exact model structure in each country application we present.

##### Susceptibility

Separate compartments are included in the model for the following categories of susceptibility to infection with *M. tuberculosis*:

- Infection-naïve
- BCG-vaccinated at birth
- Recovered following successful treatment for active disease
  - Note that spontaneous recovery from active infection is simulated as return to late latency

As the default behaviour, the latter two susceptibility categories are considered to have an equally decreased risk of infection by comparison to the infection-naïve group, although the extent of susceptibility/immunity can be varied by the user. Note that super-infection is also possible from the late latency compartment (see following section).

##### Latency

Following infection (from any susceptible or latent compartment) infected persons transition to a high-risk early latency compartment, from which they progress either to active disease or to late latency. From late latency, progression to active disease also occurs, but at a much lower rate, with super-infection also possible (with a greater degree of immunity than for the vaccinated and previously treated susceptible compartments described above). These compartments are duplicated by infecting strain and by risk group, with transition rates between these compartments equal for each strain, but potentially differing by risk groups. Progression to active disease is then split by organ involvement, as described in the following section. (This latency structure is depicted in Figure S1.)


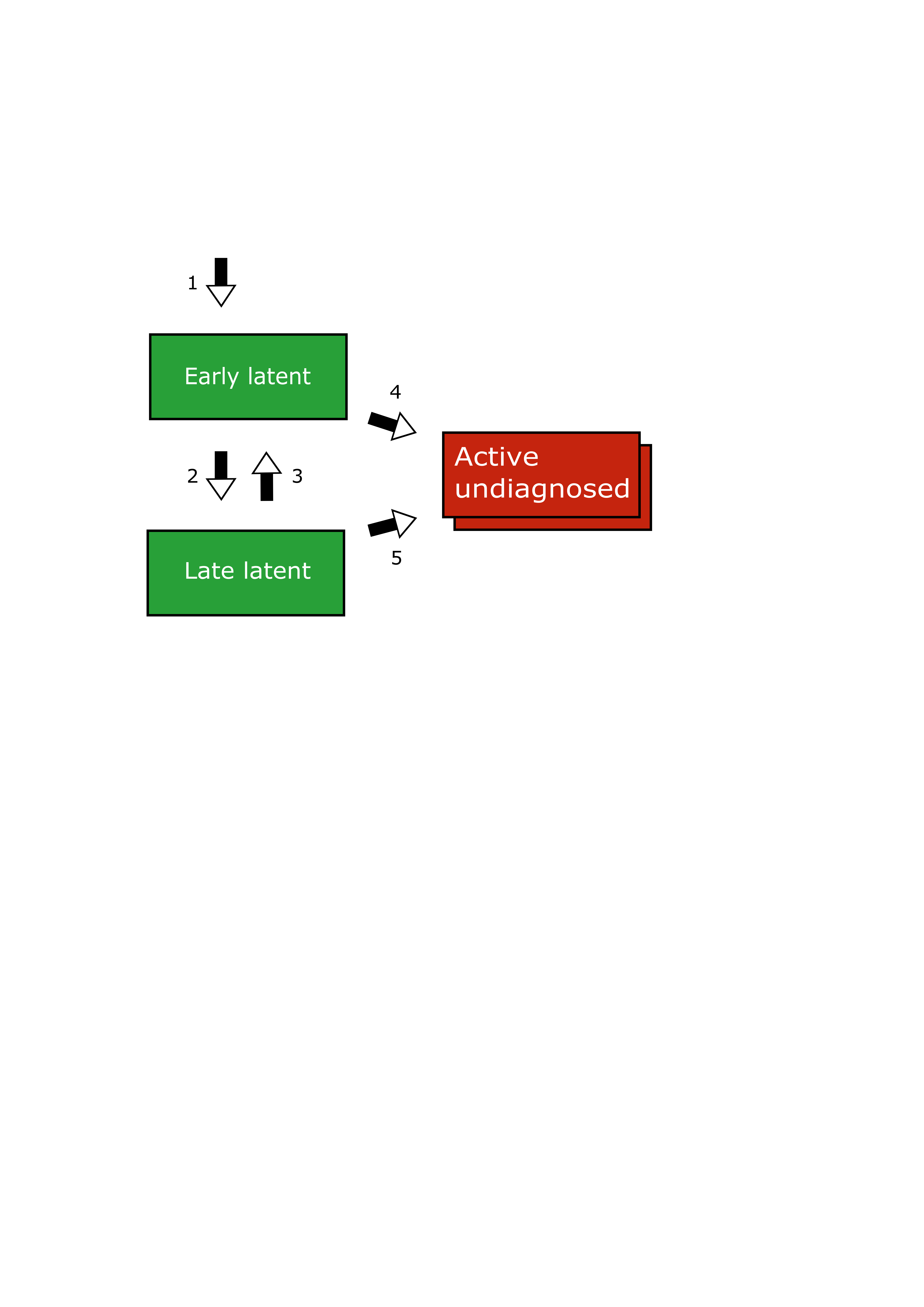


Figure S1 Compartmental latency structure. Flows presented are: 1, infection; 2, stabilisation; 3, super-infection; 4, early progression to active disease (split by organ involvement); 5, slower late progression to active disease (also split by organ involvement).

##### Progression by organ status

On progression from latency to active disease, the proportion of patients progressing to smear-positive, smear-negative and extrapulmonary TB are set equal to the proportion of notifications reported by the country in each category. That is, the total progression flow is multiplied by this time-variant, country-specific proportion that is calculated from the Global TB Report estimates of presentations by organ involvement. Extrapulmonary cases are then considered to be non-infectious, smear-positive pulmonary cases fully infectious and smear-negative pulmonary cases considered to have a reduced level of infectiousness by comparison to smear-positive pulmonary cases. (This is represented by the stacked rectangles for Active Undiagnosed cases in Figure S1.)

##### Natural history

Patients not yet commenced on a TB-specific treatment regimen are considered to spend three years with active disease before recovering or dying from their infection. That is, the flow rates out of the active disease compartments sum to one third. The case-fatality rate determines the proportion of these flows that result in death, rather than recovery, and are organ manifestation-specific (70% case fatality for smear-positive pulmonary, 20% case fatality for smear-negative pulmonary and extrapulmonary). Those spontaneously recovering return to the late latent compartment, from which reactivation and super-infection are both possible. (These transitions are represented by arrows 1 and 2 in Figure S2.)

##### Detection

The rate at which patients transition from active undiagnosed TB in the community to detection by the health system is calculated such that the proportion of all active cases that are detected is equal to the country’s reported (time-variant) case detection rate. This may also require adjustments to account for different rates of detection for smear-positive, smear-negative and extrapulmonary cases, depending on the availability of and need to simulate smear microscopy and molecular diagnostics in a particular simulation. Patients who presented for care with active TB, but for whom the diagnosis of TB was not made correctly remain in a missed compartment, such that they cannot be detected for a period of time after their presentation. As little objective evidence exists to determine the duration of this period and as it is likely to differ by context, this parameter is typically agreed in consultation with NTP staff of the country being simulated. (These transitions are represented by arrows 3 to 7 of Figure S2.)


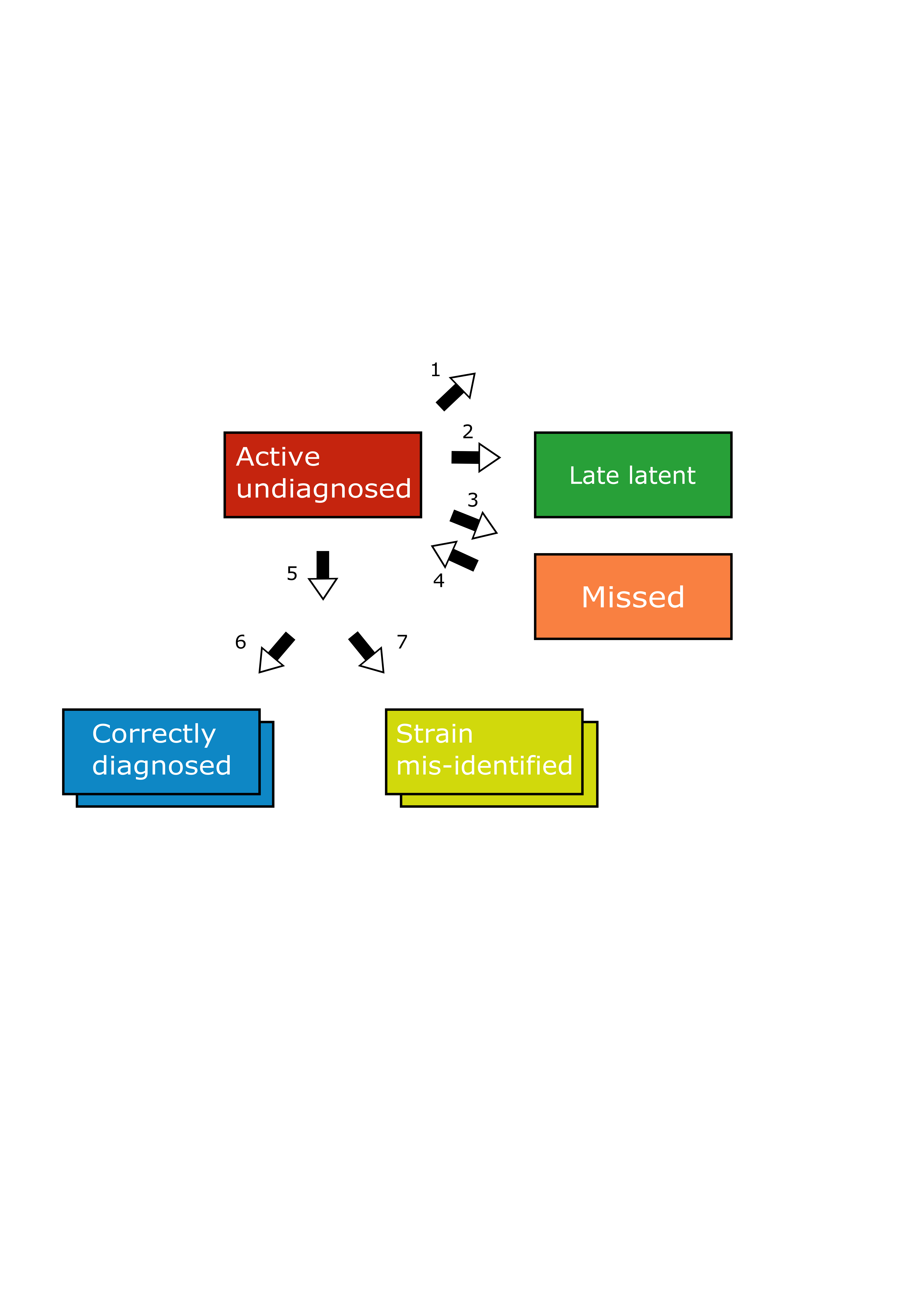


Figure S2 Natural history and detection structure. Flows presented are: 1, death untreated; 2, spontaneous recovery untreated; 3, presented to health system but TB diagnosis missed due to insufficient sensitivity of the diagnostic algorithm; 4, return to care seeking; 5, diagnosis of TB correctly made by the health system; 6, extent of drug resistance of infecting organism correctly ascertained (stratified by health system quality); 7, extent of drug resistance of infecting strain not recognised by health system (stratified by health system quality).

##### Progression through treatment

Once diagnosed on treatment, patients commence treatment depending on the availability of a treatment regimen appropriate to the drug resistance profile they were considered to have by the health system (rather than the drug resistance profile of the strain they harbour, if these differ). Patients then progress from infectious on treatment to non-infectious on treatment and finally to recovered with reduced susceptibility to re-infection as described above. The proportion of patients reaching this final recovered compartment is set equal to the (time-variant) proportion of patients reaching treatment success (cure or completion) by the country, while the proportion dying is equal to the death rate on treatment and the proportion defaulting equal to the remainder (i.e. 1 – success – death on treatment). These proportions are split between the early and late phases of treatment according to the sojourn times in these compartments. Parameters typically differ by drug resistance profile (also according to outcomes reported by the country) and patients with less drug-resistant organisms can amplify to more resistant forms with treatment default (provided their infecting strain is not already the most resistant form simulated).


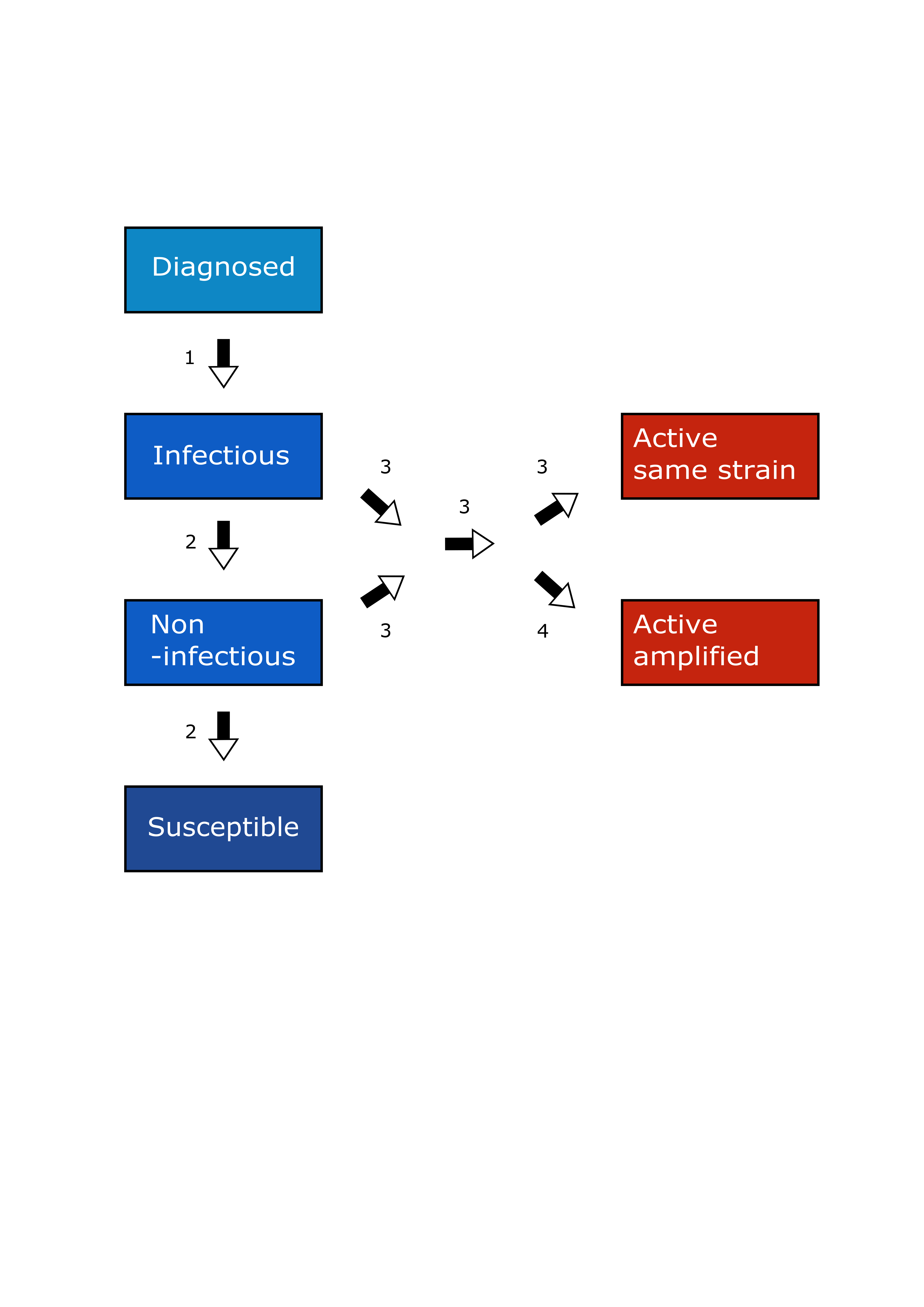


Figure S3 Compartmental treatment structure. Death under treatment flows not presented. Flows presented are: 1, commencement on treatment once appropriate regimen to identified strain becomes available; 2, progression through treatment from infectious on treatment to non-infectious on treatment to completed treatment and susceptible; 3, default from treatment; 4, amplification of drug resistance of infecting strain as a consequence of default from treatment.

### Section 2 Further example model code

| Current Python code **def set_model_with_params(self, param_dict, model_object='baseline'):**  ***"""***  ***Populates baseline model with params from uncertainty calculations.***  ***Args:***  ***param_dict: Dictionary of the parameters to be set within the model (keys parameter name strings and values***  ***parameter values).***  ***"""***  **for key in param_dict:**  **if key in self.model_dict[model_object].params:**  **self.model_dict[model_object].set_parameter(key, param_dict[key])**  **else:**  **raise ValueError('%s not in model_object params' % key)** |
| --- |
| Explanation This code defines a method to the “model runner” class that sets the newly updated set of uncertainty parameters within the model object for the following run. The model runner object holds a set of TB model objects within a dictionary attribute and has methods to run these objects for different purposes. The uncertainty parameters are updated in another method of the model runner object before being set within the model object in this method. The “**set_parameter**” method to the TB model objects is defined in the “general transmission dynamic model module”. |
| Previous closest equivalent code **for a=1:5 *%First variable varies BCG vaccination coverage***  **for b=1:5 *%Second variable varies DS-TB detection***  **for c=1:5 *%Third variable varies MDR-TB detection***  **for d=1:5 *%Fourth variable varies default rates***  **iota=0.2*(a+rand-1); *%Varies iota between zero and one***  **proptreat=ones(1,4).*(b+rand-1)/5; *%Varies delta between 50% coverage and 100%***  **mdrtreat=ones(1,4).*(c+rand-1)/5; *%Varies deltam between 50% coverage and 100%***  **omega=0.1*(d+rand-1)/td; *%Varies omega between zero and 50% default over 6 months* [TT3,y3]=wp_function(Tint,y0,mdrstart,birthstart,P,iota,proptreat,omega,**  **tdm,TT2,y2,reinfect,beta,rf);**  **end**  **end**  **end**  **end** |
| Previous closest equivalent code This line of Matlab code is also taken from our group’s first paper describing the simpler compartmental model. It specifies the values for the uncertainty parameters to be updated to and runs the model (which is a function named **wp_function**). Although this is not directly equivalent to the Python code given above, note again that the variable naming does not facilitate understanding of the meaning of the code. |

Table S1 Illustration of approach to coding 2.

### Section 3 Cost-coverage curves

A generalised logistic function is used to calculate the coverage ‘$cov$’ associated with a specific spending ‘$cost$’ on an intervention (excluding start-up costs):

$cov(cost)=A+ \frac{sat-A}{{(1+e^{-B.cost)})}^{\alpha}}$ , (1)

where $sat$ represents the saturation coverage associated with the intervention (*i.e.* the maximum coverage possible); $\alpha$ is a parameter affecting the shape of the logistic curve (equal to 1 by default); and $A$ and $B$ are constants that we determine using the conditions described below.

As start-up costs are generally considered separately from this logistic function the curve is assumed to pass through the origin, which by continuity of the logistic function implies:

$cov\left( 0 \right)=0$ . (2)

By combining equations (1) and (2), we obtain:

$A= \frac{sat}{1-2^{\alpha}}$ . (3)

From the assumption of maximal efficiency as soon as spending becomes positive (i.e. from the origin), we obtain:

$\frac{d cov}{d cost}\left( 0 \right)=\frac{1}{u.p}$ , (4)

where $u$ denotes the unit cost and $p$ the size of the population to which the intervention could potentially apply.

Deriving equation (1) and using equation (4), we finally obtain:

$B= \frac{2^{\alpha+1}}{u.p.\alpha.(sat-A)}$ .

This approach allows cost-coverage curves to be rapidly calculated during model runs from economic input parameters and model properties (i.e. sizes of the target populations).
